# Effects of Sensing Tactile Arrays, Shear Force, and Proprioception of Robot on Texture Recognition

**DOI:** 10.3390/s23063201

**Published:** 2023-03-17

**Authors:** Jung-Hwan Yang, Seong-Yong Kim, Soo-Chul Lim

**Affiliations:** Department of Mechanical, Robotics and Energy Engineering, Dongguk University, 30, Pildong-ro 1gil, Jung-gu, Seoul 04620, Republic of Korea; wjdghks9483@dgu.ac.kr (J.-H.Y.); sykim_414@dgu.ac.kr (S.-Y.K.)

**Keywords:** artificial tactile perception, tactile sensing, texture recognition, neural networks, machine leaning

## Abstract

In robotics, tactile perception is important for fine control using robot grippers and hands. To effectively incorporate tactile perception in robots, it is essential to understand how humans use mechanoreceptors and proprioceptors to perceive texture. Thus, our study aimed to investigate the impact of tactile sensor arrays, shear force, and the positional information of the robot’s end effector on its ability to recognize texture. A deep learning network was employed to classify tactile data from 24 different textures that were explored by a robot. The input values of the deep learning network were modified based on variations in the number of channels of the tactile signal, the arrangement of the tactile sensor, the presence or absence of shear force, and the positional information of the robot. By comparing the accuracy of texture recognition, our analysis revealed that tactile sensor arrays more accurately recognized the texture compared to a single tactile sensor. The utilization of shear force and positional information of the robot resulted in an improved accuracy of texture recognition when using a single tactile sensor. Furthermore, an equal number of sensors placed in a vertical arrangement led to a more accurate distinction of textures during exploration when compared to sensors placed in a horizontal arrangement. The results of this study indicate that the implementation of a tactile sensor array should be prioritized over a single sensor for enhanced accuracy in tactile sensing, and the use of integrated data should be considered for single tactile sensing.

## 1. Introduction

Artificial tactile perception that mimics human tactile perception enables delicate manipulation of robots utilizing robotic hands or grippers. Humans can accurately and quickly recognize information regarding objects, including their shape [1] or various properties such as roughness, material, and rigidity [2,3], when they come into contact with them via their the hands. For instance, when touching the surface of an object, an individual can easily discern the pattern of the fabric or the type of object being handled. This capacity for interpreting and utilizing tactile perception is an inherent aspect of human learning.

The implementation of artificial tactile perception in robots is crucial in enabling them to perform delicate manipulations when handling objects. For a robot to perform tasks based on tactile perception, it is necessary to incorporate a tactile sensor that generates tactile signals. Various types of tactile sensors, such as capacitive [4,5], piezoresistive [6,7], optical [8,9], piezoelectric [10,11], and magnetic sensors [12,13], have been developed for robots to recognize their environments during interactions by generating tactile signals. The signal generated by the aforementioned tactile sensors enables robots to execute sophisticated tasks using robot grippers that were once deemed challenging or unfeasible. These tasks include stable object grasping [14,15], slip detection [16,17], in-hand manipulation [18], and object information recognition, such as object recognition [19,20] and texture classification [21,22].

Texture recognition is a crucial task that has been the focus of various research, as it involves the estimation of one of the important surface properties of an object through contact between the sensor and the object. Drigalski et al. [23] performed classification after collecting texture data by rubbing 3-axis force tactile sensors attached to a robot gripper. Huang et al. [24] collected vibration data for textures using a single bionic tactile sensor fixed at the end of a linear motor, and performed texture classification using a convolutional neural network. Several studies have focused on collecting more comprehensive tactile data for texture recognition, rather than relying solely on pressure data. Lima et al. [25] investigated the perception of tactile textures in a two-dimensional exploration by classifying the collected data obtained using a round-shaped fingertip tactile sensor embedded with a 9-axis IMU (inertial measurement unit) sensor and barometer through machine learning. Markert et al. [21] classified the surface of objects using random forest with a concatenated power spectral density, using a 6-axis force/torque sensor attached to an end-effector as the input. Using a tactile sensor attached to one tip makes it difficult to measure the spatial features of an object along its contact path because of the narrow contact area.

Humans perceive tactile information through an ensemble of signals generated by different types of mechanoreceptors, including Merkel, Ruffini, Meissner, and Pacinian receptors in the fingertips [26,27]. The dense distribution of sensory receptors in the fingertips provides high spatial resolution for tactile perception in humans [28,29]. Several studies have been conducted on artificial tactile perception to develop tactile sensor arrays for obtaining richer tactile signals and improving spatial resolution. These include tactile sensor array based on PVDF (polyvinylidene fluoride) [30], PDMS (polydimethylsiloxane) [31], and piezoelectric materials [32]. Numerous studies have employed tactile sensor arrays for textural classification. Slepyan et al. [33] used a 3 × 3 tactile sensor array mounted on a soft biomimetic finger to distinguish 3D-printed textured plates by employing spatial frequency encoding. Gupta et al. [34] systematically disrupted the spatial arrangement of sensors for the signal output from a tactile sensor array, and subsequently proceeded with texture classification. The significant performance drop observed in the study highlights the importance of spatial information in the tactile sensor array signal for texture recognition. Several research groups have recently studied the development of a 3-axis tactile sensor array capable of measuring shear force, to obtain shear forces or skin stretch information similar to the tactile signals of the Ruffini receptors [35]. For instance, there are tactile sensors that can calculate the three-axis force by utilizing a bumped surface [36,37], and tactile sensor arrays consisting of 18 units that can measure the 3-axis force by employing point cloud fitting and triangular searching techniques [38].

In addition, tactile perception can be perceived not only by the output value of the tactile sensor but also by different types of information. Lee et al. [39] conducted a study in which they used data collected from an accelerometer and gyroscope sensors built into a smartphone as input values for a neural network to recognize the touched area on a smartphone, instead of using the tactile signal from the fingertip. Lee et al. [40] and Ko et al. [41] proposed methods that estimate the interaction force between a robot and an object while the robot grasps the object using visual images, robot position, and electrical current, without relying on tactile signals. Proprioception is necessary for tactile perception in humans. Dysfunctions in proprioception can lead to difficulties in accurately judging the shape of the object being touched and the distance to the point of contact [42] and difficulties in perceiving the surface roughness of an object [43]. For artificial tactile perception, it is necessary to study the effect of robot positional information and investigate strategies to utilize tactile information in the target task.

Although several studies have been conducted to implement artificial tactile sensing, achieving the level of accuracy in performing tasks through tactile information that humans possess still remains a challenge. Several factors influence tactile perception. However, previous studies on tactile sensor arrays mainly focused on improving sensor performance and sensitivity, with limited research on the impact of various factors on artificial tactile perception. To achieve effective tactile perception, it is necessary to investigate the effects of tactile sensing arrays, shear force, and robot positional information on task performance.

In this study, we focused on investigating the effects of tactile sensor arrays, shear force, the positional information of the robot, and integration of these types of tactile data on texture recognition through deep learning. A simple tactile sensing system was established to collect the tactile data. The system includes a tactile sensor array that can measure the normal force, a force-torque sensor capable of detecting shear force, and a robot arm that performs object exploration. Using this system, the time-series tactile sensor data and robot positions were collected for 24 textures. By varying the input values of the same deep learning network, the type of information that affects artificial tactile perception was investigated. To enhance the precision of artificial tactile perception, it is recommended to use a tactile array sensor instead of a single sensor. When using a single tactile sensor, it was observed that the texture recognition accuracy was improved by additionally utilizing the shear force and robot position information. Additionally, the accuracy of a tactile sensor array with the same number of sensors can vary based on the arrangement. Identifying the factors that affect artificial tactile perception and using appropriate tactile information can aid robots in performing target tasks accurately.

## 2. Materials and Methods

A simple tactile sensing system was constructed to investigate the effects of sensing the tactile array, shear force, and robot positional information on texture classification. In the tactile sensing system, a robot gripper was attached to the end of a 6 degrees of freedom (6-DoF) robot arm. Each fingertip of the robot gripper has a built-in force-torque sensor. The tactile sensor array was located above each fingertip of the gripper. The robotic arm examined 24 types of textures to collect tactile data. A deep learning algorithm was employed to classify the textures.

### 2.1. Texture Exploration Interface and Tactile Sensor Array

**Figure 1:**
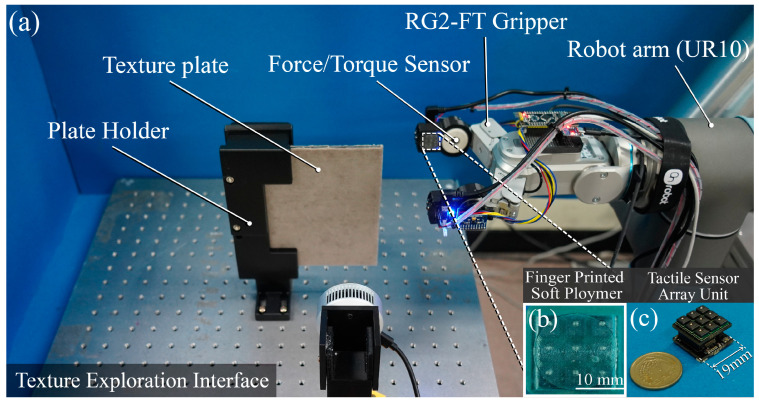
Used texture exploration interface: (**a**) The tactile sensing system. A 3 × 3 tactile sensor array was attached to each end of the gripper (RG2-FT, OnRobot) equipped with force-torque sensors. The robot arm (UR-10, Universal Robots) was used for texture exploration. (**b**) A tactile sensor array covered with fingerprinted soft polymer. The soft polymer made of elastic resin (Engineering Resin, Formlabs) with a shore hardness of 50 A has a fingerprint’s ridge depth of 0.25 mm, a width thickness of 0.1 mm, and a gap between the ridge of 0.2 mm. (**c**) Photographs of the tactile sensor array module with a coin. The size of the tactile sensor array is 19 mm × 17 mm.

Figure 1a shows our tactile experimental interface, comprising a robot gripper (RG2-FT, OnRobot, Odense, Denmark), robot arm (UR-10, Universal Robots, Odense, Denmark), and a tactile texture plate fixed by a 3D-printed holder on the xy plane. The robotic arm performed exploration along the z-axis (elevation) in the vertical direction of the experimental plane. The tactile sensor array was located on both sides of the robot gripper fingertips, and the height of the contact surface remained unchanged; thus, the robot gripper and tactile sensor array could simultaneously come into contact with the object.

In this study, a 3 × 3 tactile array sensor consisting of nine FMA MicroForce Sensors (FMAMSDXX005WCS3, Honeywell, Charlotte, NC, USA) was used. The FMA MicroForce Sensor is a piezoresistive force sensor that provides a digital output proportional to the applied force. The tactile sensor is available in different versions with various force ranges, including 5, 15, and 25 N. In the current study, the 5 N normal force range with a 0.1 N accuracy and an overforce limit of 15 N was used. Each 3 × 3 tactile sensor array module comprises nine tactile sensors; therefore, we opted to use SPI communication (four wire) instead of I2C communication, which has a limited address availability. The sensor has a typical response time of 0.42 ms, and SPI communication allows for a higher digital clock frequency of up to 800 kHz, and can be powered by an operating voltage of 3.3 V. The dimensions of the sensor are 5 mm × 5 mm. The FMA MicroForce Sensors were arranged in a 3 × 3 configuration and mounted onto a printed circuit board (PCB), as illustrated in Figure 1c. The size of the tactile sensor array module, which included nine tactile sensors, is 19 mm × 17 mm. The tactile sensor array measures the normal force at 500 Hz.

We covered the surfaces of the tactile sensor array modules with a soft polymer-printed fingerprint (Figure 1b) based on a human fingertip. The soft polymer was printed using a 3D resin printer (Form3, Formlabs, Sommerville, MA, USA). The soft cover material was an elastic resin (Engineering Resin, Formlabs, Sommerville, MA, USA) with a shore hardness of 50 A. Considering the durability of the soft cover’s fingerprint and the technical ability of the printer, the fingerprint’s ridge depth on the softcover, the width thickness, and the gap between the ridge were calculated to be 0.25, 0.1, and 0.2 mm, respectively. The size of the tactile sensor array module with the soft polymer cover was 20 mm × 20 mm. By covering it with a fingerprinted polymer, the tactile sensor array can efficiently detect the frequency of the haptic response [44]. In addition, the tactile force generated by the interaction between the sensor and the object was evenly distributed to each individual tactile sensor. Linear regression was employed to calibrate the tactile sensor array using a force-torque sensor (ATI Mini40 force/torque sensor, ATI Industrial Automation, Apex, NC, USA).

### 2.2. Experimental

**Figure 2:**
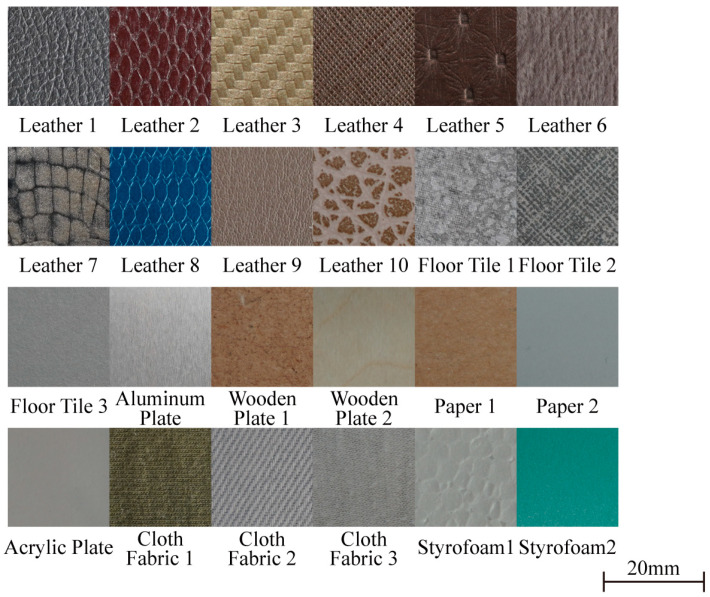
Twenty-four types of textures used for the experiment, including ten types of leather, three types of ceramic floor tiles, an aluminum plate, two types of wooden plate, two types of paper, an acrylic plate, three types of cloth fabric, and two types of Styrofoam. These 24 textures were selected based on their surface pattern, hardness, roughness, and friction. Each image corresponds to a 20 mm × 20 mm area of the texture surface. A scale bar of 20 mm is displayed in the lower left corner.

Figure 2 shows the 24 textures used in this study. The textures used in the experiment consisted of a variety of materials, including four types of patterned artificial leather (herringbone pattern, saffiano pattern, dot pattern, and crunch pattern), six types of animal leather (rhino, two types of snakes, alpaca, crocodile, and cow), three types of floor tile (a rough surface, a relatively smooth but uneven surface, and a large pattern), an aluminum plate, two types of wooden plate (rough medium density fiberboard and smooth plywood), two types of paper (packing box and A4 paper), acrylic plates, three types of cloth fabric (denim, soft fabric, and cotton), and two types of Styrofoam with different particle sizes. The size of floor tiles 1–3, the aluminum plate, wooden plate 1, and wooden plate 2 were 25 cm × 25 cm, while the remaining texture plates were 15 cm × 15 cm. Textures, except for the wooden plates, the aluminum plate, floor tiles 1–3, the acrylic plate, and paper 1 (the part of a packing box), were stuck to both sides of an acrylic plate with a thickness of 3 mm.

**Figure 3:**
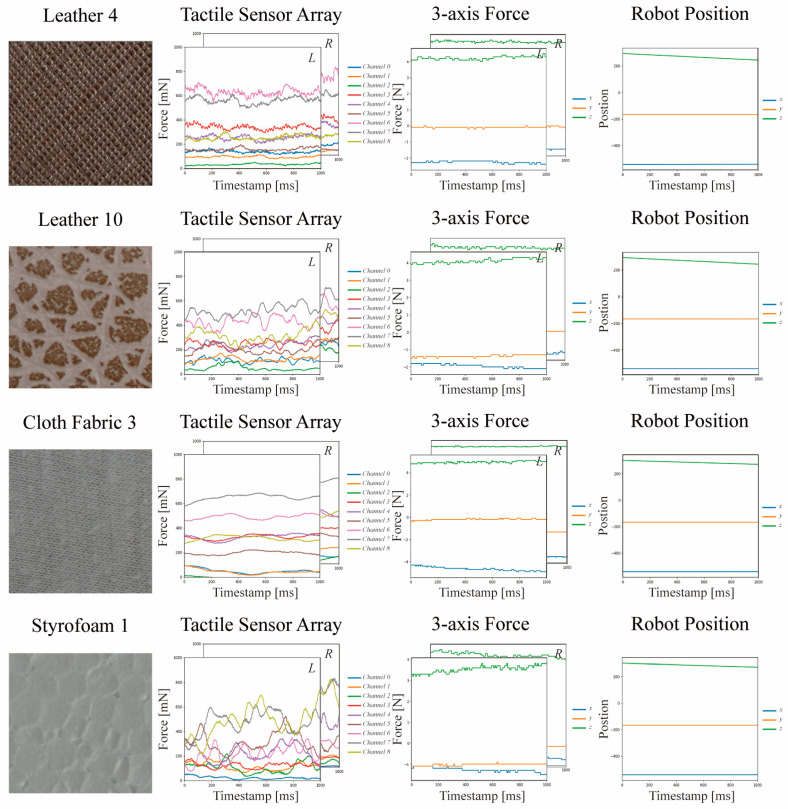
Examples of tactile surface exploration data for four textures (leather 4, leather 10, cloth fabric 3, and Styrofoam 1) out of the total twenty-four textures collected within the tactile sensing system. From left to right, the data were collected using a 3 × 3 tactile sensor array with nine channels, 3-axis force data, and robot position data.

Tactile data were collected by scanning vertically below the tactile texture plates, which were positioned at the center of the experimental interface. Figure 3 shows an example of tactile surface exploration data for four textures (leather 4, leather 10, cloth fabric 3, and Styrofoam 1) out of the total 24 textures collected by the tactile sensing system. The complete dataset of tactile information comprised the data collected from both tactile sensor arrays, the two sets of 3-axis force data obtained from the force-torque sensor, and the positional information of the robot arm’s end-effector in terms of its x, y, and z coordinates. The sampling rate for each data point was 500 Hz for the 3 × 3 tactile array sensor, 250 Hz for the force-torque sensor, and 125 Hz for the robot position. Owing to the difference in sampling rates, the data were synchronized by storing at 1 kHz.

Considering the differences in the tactile sensor data based on the exploration speed, texture exploration was conducted at 20, 30, 50, and 70 mm/s. The experiment was conducted as follows: The tactile data collection process involved the robotic arm moving towards the texture plate, grasping the top portion of the plate with the gripper, and exploring downwards at a constant velocity. During this process, the tactile sensor array, the 3-axis force data from the force-torque sensor on both sides of the fingertips, and the positional coordinates of the end-effector were recorded. The exploration stopped after the robot arm moved 100 mm downward from the exploration start point and the gripper opened. The robot arm then returned to its original position, and this process was repeated 18 times. The experiment was then repeated with different velocities for exploration. The data collection procedures were automated, and a total of 1728 trials were conducted, involving 24 types of texture plates, 4 velocities, and 18 sets.

For classification, the data were cropped to unify the data length per set. Instead of using the tactile data from the beginning and end of the exploration time, where a change in velocity existed, we utilized the data collected from −500 ms to +500 ms from the center of the exploration data. The training and test data consisted of the front part of each set (0 ms to 700 ms) and the rear part of each set (700 ms to 1000 ms), respectively.

### 2.3. Deep Learning Network

Various methods, such as the support vector machine [45], K-nearest neighbor (KNN) [46], convolution neural network (CNN) [47], fast Fourier transform (FFT) [48], and recurrent spiking neural network [49], have been suggested for texture recognition. Long short-term memory (LSTM) [50] has been widely used in a several tasks for processing time-series data [51], such as trajectory prediction [52], acoustic modeling [53], and speech recognition [54]. Considering that the collected tactile data were time-series data, an LSTM-based network was used in this study. The network used for texture classification was the same for investigating the effects of the sensing tactile array, shear force, and robot information, with the exception of the input values provided to the network.

**Figure 4:**
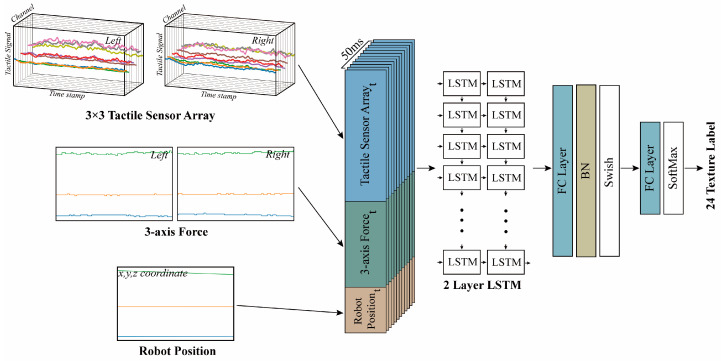
Neural network architecture used for the classification of 24 textures. The input to the network consists of 3 × 3 tactile sensor arrays (left and right), 3-axis force data (left and right), and robot position information. Sequence data cropped at 50 ms intervals (window length) are concatenated and fed into the LSTM layer. The 64 features generated from the two LSTM layers are finally processed through a fully connected layer (FC layer), resulting in the classification of 24 textures.

Figure 4 shows the neural network structure used in this study. The inputs were tactile data collected over 50 ms, which were randomly cropped from the continuous set using a window with a length of 50. The tactile data were selected or concatenated from the values of tactile sensor arrays, 3-axis force, and robot positional information, depending on the purpose of the investigation. The network was designed to perform texture classification using softmax as the final layer. The output of the network provided the probability distribution of the input data belonging to one of the twenty-four texture classes. The network consisted of two LSTM layers and two dense fully connected layers. The LSTM layers received n (measurements) × 50 inputs. The measurements consisted of tactile sensor arrays (number of tactile units used × two fingertips), 3-axis force (n = three axes × two fingertips), and robot position (n = 3 x, y, and z coordinates). If multiple types of data were utilized as input, they were concatenated together. The last LSTM layer outputted 64 features from the last LSTM cell. In the last LSTM cell, the output the of 64 features was fed into the first fully connected layer. The output of the 48 neurons from the first fully connected layer was passed through batch normalization and Swish activation [55] before being forwarded to the second fully connected layer. After the second fully connected layer, 24 neurons were output, each representing one of the 24 textures. Finally, softmax was applied to classify the input into one of the twenty-four texture categories. During model training, a batch size was 4096 was used, and the Adam optimizer [56] was utilized. The initial learning rate was 0.001 and the LambdaLR scheduler for decaying the learning rate by a factor of 0.95 was used for every epoch. Cross-entropy was used as the loss function. The model was trained for up to 100 epochs on an NDIVIA TITAN Xp GPU. The results with the highest accuracy were selected after learning and testing each input value three times.

## 3. Results

### 3.1. Effect of Sensing Tactile Array on Texture Recognition

**Figure 5:**
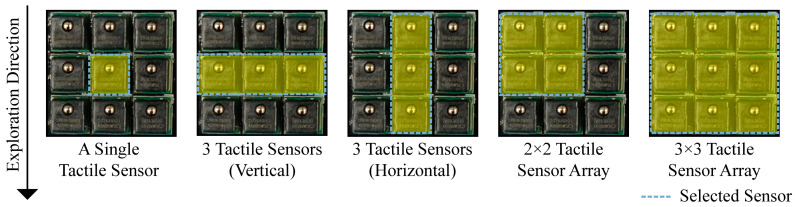
Tactile sensors used as inputs to the neural network for investigating the effect of sensing tactile array on texture recognition. With single tactile sensing, the data from one sensor located at the center of a 3 × 3 tactile sensor array were used. To investigate the effect of the arrangement of sensors on texture recognition using the same number of tactile sensors, sensors were used consisting of three vertical sensors passing through the center and three horizontal sensors passing through the center. The sensor located on the remaining side of the gripper was also used in the same manner.

**Figure 6:**
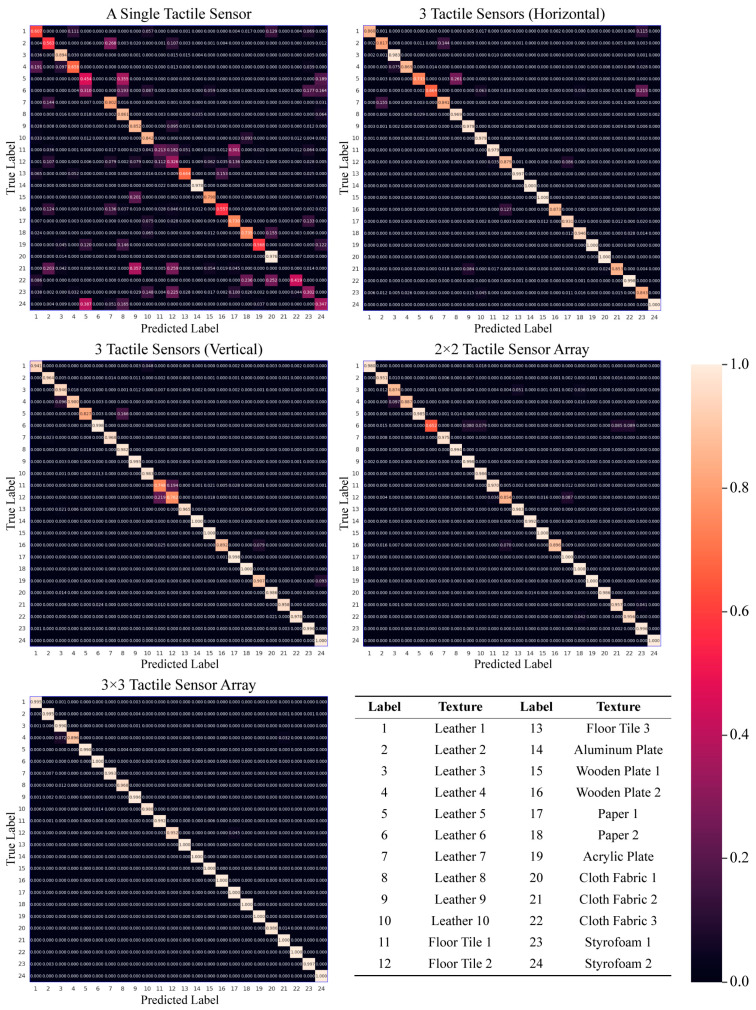
Confusion matrix of texture classification to compare the effects of sensing tactile arrays. The average accuracy for a total of 24 textures, represented by the main diagonal elements of the confusion matrix, is 58.913% for a single tactile sensor, 91.648% for three tactile sensors (horizontal), 94.572% for three tactile sensors (vertical), 95.292% for a 2 × 2 tactile sensor array, and 98.876% for a 3 × 3 tactile sensor array. The color bar located at the bottom right indicates the degree of accuracy, with lighter colors indicating higher accuracy and darker colors indicating lower accuracy. The order of textures in the true label and predicted label of the four confusion matrices corresponds to the order of textures listed in Figure 2.

We compared the texture classification performance based on the number of tactile sensors and their arrangement to examine the effects of the sensing tactile array. Single sensing (at the center of the tactile array sensor), three tactile sensors (vertical and horizontal in the direction of exploration), 2 × 2 tactile sensor arrays, and all 3 × 3 tactile sensor arrays located on both sides of the fingertip were used as the input values for the deep learning network (Figure 5). Table 1 shows the accuracy results of the texture classification according to the number and arrangement of tactile sensors. Figure 6 shows the confusion matrix for texture classification. The order of the texture labels on the x-axis (predicted label) and y-axis (true label) of the confusion matrix is the same as that shown in Figure 2 and can also be verified in the bottom left of Figure 6. The results revealed that the texture classification accuracy was affected by the number of tactile sensors. Specifially, the accuracy was observed to increase with a higher number of tactile sensors. The accuracy of a single sensor (at the center of the tactile sensor array) was 58.913%, which was the lowest among the results. In the case of single sensing, the textures of leather 6 and cloth fabric 2 were not recognized, and it was difficult to distinguish the textures, with the exception of aluminum and cloth fabric 1, which were confused with the others.

The results showed accuracies of 94.572% for the vertical arrangement and 91.648% for the horizontal arrangement, which were over 32% higher than the accuracy achieved with a single sensor. The study found that the arrangement of the tactile sensors during exploration had an impact on the texture classification accuracy. When exploring in the vertical direction, the arrangement of the sensors resulted in a 2.924% higher accuracy than the horizontal arrangement. However, the vertical arrangement resulted in confusion between floor tiles 1 and 2, which had hard and bumpy surfaces. Conversely, the horizontal arrangement classified floor tiles 1 and 2 with higher accuracy than the vertical arrangement, but had difficulty classifying leather 5, which had a dot pattern, leather 6, which had no pattern, and soft fur compared to the vertical arrangement. The 2 × 2 tactile sensor array showed a higher texture classification accuracy of 95.292% compared to the use of three tactile sensors. Leather 6, which was poorly classified by three tactile sensors (horizontal), remained to be challenging to classify. However, Leather 5, which was poorly classified with three tactile sensors (horizontal), was classified with a higher accuracy using the vertical and horizontal arrays of three tactile sensors. Moreover, floor tiles 1 and 2, which were poorly classified with three tactile sensors (vertical), were classified with a higher accuracy than the use of three tactile sensors (vertical) and a lower accuracy than the use of three tactile sensors (horizontal). The use of the tactile sensor array with nine sensors per fingertip resulted in the highest accuracy of 98.876%.

The classification accuracies of the 3-axis force and tactile sensor array were also compared. The accuracy (95.292%) of the 2 × 2 tactile sensor array and the accuracy (98.876%) of the 3 × 3 tactile sensor array, which could detect normal force, were higher than that of the 3-axis single force sensing (95.08%) (Table 2). The study found that despite the tactile sensor array being able to detect only normal force, it was still more effective in classifying the textures used in the experiment compared to the three-axis single sensing.

### 3.2. Effect of Sensing Shear Force on Texture Recognition

Various tactile information is used when a person interacts with an object. Shear force also has a significant effect on tactile perception. For example, when shear and normal forces are sensed simultaneously during dynamic movement, the frictional characteristics of an object can be measured. Therefore, we investigated how the addition of shear force information to normal force affects texture classification.

The tactile sensor array used in this study could only measure the normal force. Therefore, we concatenated the shear force value of the force-torque sensors and the value of the tactile sensor array to determine the effect of shear force sensing. The concatenated data were provided as the input values for the deep learning network. Adding shear force data improved the classification accuracy compared to using only the tactile sensor array data. The integrated data, which combined the normal force values from the tactile sensor array and the shear force values from the 3-axis single force sensing, improved the classification accuracy of all textures, including leather 4, which had an accuracy of less than 90% when using only the tactile sensor array. The accuracy of concatenated data with shear force and a single tactile sensor was 96.842%, which was 37.929% higher than using a single tactile sensor alone (58.913%), as shown in Table 3. These results indicate that it is necessary to consider the shear force to accurately recognize the texture.

Although both acquired texture information through 3-axis single sensing, the accuracy of the concatenated data of a single tactile sensor and shear force was 1.762% higher than that of 3-axis force. This result was obtained because the sampling rate of the single tactile sensor measuring the normal force was higher than that of the 3-axis force sensor.

### 3.3. Effect of Tactile and Proprioception Information on Texture Recognition

Proprioceptive and tactile information were combined for roughness perception [43] and shape discrimination [57] during the exploration of an object using a human finger. We investigated the effect of the robot position on texture recognition. Table 3 shows a comparison of the results of the proprioceptive effects during the robot’s exploration of objects.

The concatenated data of the tactile sensor array and the x, y, and z coordinates of the end effector were used as input values for the deep learning network. Using the integrated data, the textures were classified with an accuracy of 99.303%, which was higher than when only the tactile sensor array data were used. Similar to the results in the previous section (the effect of shear force), all textures were classified with over 90% accuracy. When the positional information of the robot was included, the accuracy of single sensing improved by 28.909% (Table 3). These results demonstrate that the robot positional information increases the accuracy of artificial tactile perception.

Furthermore, we examined how the combination of tactile signals from a tactile sensor array and a 3-axis force sensor with the position of the robot affects accuracy, as combined tactile and proprioceptive information is required for human tactile perception. The concatenate with the tactile sensor array, the 3-axis force, and the coordinate of the end effector were provided as the input to the neural network. The result of texture classification using this combined data was 99.790%, which is the highest accuracy result obtained in this study. When concatenated data with a single tactile sensor, 3-axis force, and robot positional information were input into the network, the accuracy of texture classification was 98.425%, which was the highest accuracy of the input values using a single tactile sensor.

However, even when single tactile sensor, 3-axis force sensor, and robot position information were used, the accuracy of the texture classification was lower than the accuracy of a tactile sensor array. This demonstrated the importance of using a tactile sensor array for accurate tactile perception. However, this also indicates that single tactile sensing data combining shear force and robot position information can achieve tactile recognition as accurately as a tactile array sensor.

## 4. Discussion

Human tactile perception is influenced by various factors, such as four types of mechanoreceptors [26,27], proprioception [43,57], and visual data [58]. We conducted 24 texture classifications to evaluate the impact of the array sensor, shear force, and robot positional information on artificial tactile perception. The accuracy of texture classification based on the number of tactile sensors was compared to investigate the effect of a sensing tactile array on tactile perception. The accuracy decreased in the order of the 3 × 3 tactile sensor array, three tactile sensors, and the single tactile sensor (Table 1). Similar to the high concentration of mechanoreceptors in human fingertips [28], which enables outstanding texture recognition, the results of this study indicate that rich signals acquired from several tactile sensors and the improved spatial resolution through the arrangement of tactile sensors can accurately represent texture information in artificial tactile perception.

Moreover, Table 1 shows that there is a difference in the accuracy of texture recognition resulting from the arrangement of tactile sensors. With the same number of tactile sensors, the accuracy of the vertical arrangement for object exploration was higher than that of the horizontal arrangement. The vertical arrangement scans the surface of an object with a wider range than the horizontal arrangement; therefore, it can be concluded that the vertical arrangement detects the spatial features of the texture better than the horizontal arrangement. The spatial features of the texture were more helpful for texture recognition than the temporal features detected by overlapping searches of the same part of the texture.

After searching the same texture area, the accuracy of the three tactile sensors placed horizontally in the search direction was found to be significantly higher than that of the single tactile sensor. This indicates that the temporal features of the textures obtained by overlapping scans with short time intervals are useful for texture classification. Additionally, because the tactile sensors were covered with a soft polymer that had a fingerprint pattern, the tactile signals detected in the same area of the surface were found to be represented differently and transmitted to each tactile sensor. This differential representation of tactile signals could enhance tactile recognition and underscores the significance of fingerprinted soft covers in artificial tactile perception.

The 2 × 2 tactile sensor array performed better than both versions of the three tactile sensors in terms of accuracy. Nevertheless, the confusion matrix showed that a 2 × 2 tactile sensor array is not necessarily better than three tactile sensors for all textures (Figure 6). Leather 5, which was poorly distinguished by the three tactile sensors (horizontal), was also poorly recognized by the 2 × 2 array. However, three tactile sensors (vertical) performed well in distinguishing leather 5. This suggests that the 2 × 2 tactile sensor array has a limitation in recognizing the spatial features of textures as it explores a smaller area than the three tactile sensors (vertical). Furthermore, the 2 × 2 tactile sensor array showed higher accuracy in distinguishing floor tile 1 and floor tile 2 compared to the three tactile sensors (vertical), but a lower accuracy compared to the three tactile sensors (horizontal). It can be inferred that the 2 × 2 array tactile sensor can detect temporal and spatial features of textures better than three tactile sensors (vertical and horizontal) on average, but there is a limit to recognizing the features of texture because the number of tactile sensors constituting the array is not sufficient.

Roughness helps to distinguish different textures [59]. The sensation of roughness is affected not only by the normal force but also by the shear force and friction [60]. The friction coefficient is defined as the shear force over the normal force, and it is necessary to measure the shear force for accurate recognition of textures. Using a single 3-axis force sensor, the accuracy of texture classification was found to be higher than that of a single normal tactile sensor (Table 1 and Table 2). A single normal sensor could distinguish textures based on the differences in the macro features of the surfaces; however, it was difficult to distinguish textures with similar surface patterns but different frictional properties. Conversely, single 3-axis force sensing can measure both normal and shear forces simultaneously; therefore, the texture can be distinguished by the difference in the macro-pattern and the friction of the texture. The accuracy of texture classification using a single 3-axis force sensor was higher than the accuracy of three normal tactile sensors. This study confirmed the importance of sensing the friction force of an object through shear force measurements for texture recognition. This result does not imply that a single 3-axis force sensor is more suitable for texture classification than a tactile sensor array. The classification accuracy using single 3-axis force sensing was lower than the accuracy of the tactile sensing array used in this study. The reason for this is that single sensing cannot capture the temporal features of textures obtained through overlapping scans or the fine spatial features of textures sensed at a higher spatial resolution.

Shear force data were added to the tactile sensor information to sense the fine surface features and friction of the textures. The accuracy was higher than that of the measurements obtained using only the tactile sensor array and a single tactile sensor. This indicates that there is a limit to the expression of texture as a normal force, and that the shear force for sensing the friction of texture is required for accurate texture recognition. Similar to the vital role that Ruffini receptors play in detecting shear force in human tactile perception [35], the significance of incorporating shear force in artificial tactile sensing has also been established.

In human perception, the roughness of a texture can be accurately recognized when it is scanned through hand movements, even when the scanning speed and contact force vary. This is possible because proprioceptive information allows the brain to perceive the search position and speed [43]. We applied this concept to artificial tactile perception and compared the results of our experiments with and without the coordinates of the end effector of the robot, which can determine the scanning speed and position. The accuracy of texture classification was increased by providing positional information about the robot (Table 3). This improvement in accuracy indicates that providing the robot position could assist in finding correlations between the pattern of texture and the scanning position or perceiving signal changes at the four exploring speeds. The exploration direction and speed can be implied through robot positional information; therefore, using the x, y, and z coordinates of the end effector with tactile signals enables object recognition with higher accuracy. This indicates that artificial tactile perception is not only related to tactile signals, but also to indirect information such as the coordinates of the end effector. In addition, in single sensing, the utilization of shear force information is more effective than using the robot position. However, the accuracy of texture recognition was similar when using both the tactile sensor array and robot position information, or both the tactile sensor array and shear force information. This result suggests that a using tactile sensor array with robot position information, which does not require the purchase of expensive force-torque sensors or the installation of force-torque sensors in tactile systems, is an efficient way to improve texture recognition accuracy.

In the final experiment, the use of integrated data, which consisted of the tactile sensor array, shear force, and positional information of the end effector, resulted in the highest accuracy in classifying textures. Based on the results, while using a tactile sensor array with 3-axis force and robot position information numerically showed the highest accuracy in texture recognition, the difference in accuracy between adding only shear force information and both 3-axis force and robot position information was negligible. Therefore, utilizing both types of information for texture recognition in tactile sensing arrays is ineffective. However, with the use of single sensing, a stack of these measures clearly showed an improvement in tactile perception. This approach is a cost-effective and straightforward manufacturing process that employs single sensors and electronic circuits. Our results demonstrate that, similar to the complexity of human tactile perception, artificial tactile perception is influenced by multiple factors. Therefore, for precise artificial tactile perception, single tactile sensing and other parameter measurements should be employed.

## 5. Conclusions and Future Work

This study investigated the impact of a normal tactile array, shear force, and robot positional information on tactile classification. Furthermore, real-time texture recognition was demonstrated in the texture exploration interface (Video S1). Our findings suggest that a tactile sensor array provides a higher accuracy of texture classification than a single tactile sensor, and the configuration of the tactile sensor array also influences the texture classification. This highlights the importance of utilizing a tactile sensing array rather than a single tactile sensor in artificial tactile perception and provides insight into the arrangement of the tactile sensors for efficient tactile recognition. Furthermore, our study showed that combining a single tactile sensor with 3-axis force and the position of the end effector can improve texture recognition, although the use of a tactile array sensor and all information did not yield a significant effect. As humans use multiple senses for tactile perception, factors other than tactile sensors have been demonstrated to impact artificial tactile perception. Therefore, various complex measurements and a single tactile sensor can be utilized for effective artificial tactile sensing.

In future studies, we plan to develop a tactile sensor array that includes a greater number of tactile sensors and investigate the impact of the arrangement of tactile sensors on tactile perception for the same number of tactile sensors. Moreover, we aim to incorporate integrated measurements that have an impact on artificial tactile sensing to perform specialized tasks that require a deeper understanding of tactile perception, such as in-hand manipulation.

## Figures and Tables

**Table 1:** Accuracy results of texture classification according to the number of tactile sensors.

Number of Tactile Sensors	Accuracy (%)
3 × 3 Tactile Sensor Array	98.876
2 × 2 Tactile Sensor Array	95.292
Three Tactile Sensors (Vertical)	94.572
Three Tactile Sensors (Horizontal)	91.648
A Single Tactile Sensor	58.913

**Table 2:** Accuracy results of texture classification of a single 3-axis force sensor built into the robot gripper.

Input of Neural Network	Accuracy (%)
3-axis force	95.080

**Table 3:** Accuracy results of texture classification of tactile sensor concatenated with shear force and robot position.

Concatenate	Accuracy (%)	
	A Single Tactile Sensor	3 × 3 Tactile Sensor Array
3-axis force and robot position	98.425	99.790
Shear force	96.842	99.396
Robot position	87.822	99.303
-	58.913	98.876

## Data Availability

Data sharing not applicable.

## Supplementary Materials

The following supporting information can be downloaded at: https://www.mdpi.com/article/10.3390/s23063201/s1, Video S1: Demonstration of texture recognition.

## References

[1] Harmon L.D. (1982). Automated tactile sensing. Int. J. Robot. Res..

[2] Manfredi L.R., Saal H.P., Brown K.J., Zielinski M.C., Dammann J.F., Polashock V.S., Bensmaia S.J. (2014). Natural scenes in tactile texture. J. Neurophysiol..

[3] Fagiani R., Massi F., Chatelet E., Berthier Y., Akay A. (2011). Tactile perception by friction induced vibrations. Tribol. Int..

[4] Taunyazov T., Koh H.F., Wu Y., Cai C., Soh H. Towards effective tactile identification of textures using a hybrid touch approach. Proceedings of the 2019 International Conference on Robotics and Automation (ICRA).

[5] Pagoli A., Chapelle F., Corrales-Ramon J.-A., Mezouar Y., Lapusta Y. (2022). Large-Area and Low-Cost Force/Tactile Capacitive Sensor for Soft Robotic Applications. Sensors.

[6] Erukainure F.E., Parque V., Hassan M.A., FathElbab A.M. Towards estimating the stiffness of soft fruits using a piezoresistive tactile sensor and neural network schemes. Proceedings of the 2022 IEEE/ASME International Conference on Advanced Intelligent Mechatronics (AIM).

[7] Gupta N., Adepu V., Tathacharya M., Siraj S., Pal S., Sahatiya P., Kuila B.K. (2023). Piezoresistive pressure sensor based on conjugated polymer framework for pedometer and smart tactile glove applications. Sens. Actuators A Phys..

[8] Taylor I.H., Dong S., Rodriguez A. GelSlim 3.0: High-resolution measurement of shape, force and slip in a compact tactile-sensing finger. Proceedings of the 2022 International Conference on Robotics and Automation (ICRA).

[9] Trueeb C., Sferrazza C., D’Andrea R. Towards vision-based robotic skins: A data-driven, multi-camera tactile sensor. Proceedings of the 2020 3rd IEEE International Conference on Soft Robotics (RoboSoft).

[10] Kumaresan Y., Ma S., Shakthivel D., Dahiya R. AlN Ultra-Thin Chips Based Flexible Piezoelectric Tactile Sensors. Proceedings of the 2021 IEEE International Conference on Flexible and Printable Sensors and Systems (FLEPS).

[11] Luo J., Zhang L., Wu T., Song H., Tang C. (2021). Flexible piezoelectric pressure sensor with high sensitivity for electronic skin using near-field electrohydrodynamic direct-writing method. Extrem. Mech. Lett..

[12] Yan Y., Shen Y., Song C., Pan J. (2022). Tactile Super-Resolution Model for Soft Magnetic Skin. IEEE Robot. Autom. Lett..

[13] Bhirangi R., Hellebrekers T., Majidi C., Gupta A. (2021). ReSkin: Versatile, replaceable, lasting tactile skins. arXiv.

[14] Li T., Shu X., Yang K., Wu C., Chen G. Robot Grasping Stability Prediction Network based on Feature-fusion and Feature-reconstruction of Tactile Information. Proceedings of the 2022 IEEE International Conference on Mechatronics and Automation (ICMA).

[15] Liu H., Huang B., Li Q., Zheng Y., Ling Y., Lee W., Liu Y., Tsai Y.-Y., Yang C. Multi-fingered Tactile Servoing for Grasping Adjustment under Partial Observation. Proceedings of the 2022 IEEE/RSJ International Conference on Intelligent Robots and Systems (IROS).

[16] James J.W., Lepora N.F. (2020). Slip detection for grasp stabilization with a multifingered tactile robot hand. IEEE Trans. Robot..

[17] Sui R., Zhang L., Li T., Jiang Y. (2021). Incipient Slip Detection Method With Vision-Based Tactile Sensor Based on Distribution Force and Deformation. IEEE Sens. J..

[18] Lambeta M., Chou P.-W., Tian S., Yang B., Maloon B., Most V.R., Stroud D., Santos R., Byagowi A., Kammerer G. (2020). Digit: A novel design for a low-cost compact high-resolution tactile sensor with application to in-hand manipulation. IEEE Robot. Autom. Lett..

[19] Pohtongkam S., Srinonchat J. (2021). Tactile Object Recognition for Humanoid Robots Using New Designed Piezoresistive Tactile Sensor and DCNN. Sensors.

[20] Kirby E., Zenha R., Jamone L. (2022). Comparing single touch to dynamic exploratory procedures for robotic tactile object recognition. IEEE Robot. Autom. Lett..

[21] Markert T., Matich S., Hoerner E., Theissler A., Atzmueller M. Fingertip 6-Axis Force/Torque Sensing for Texture Recognition in Robotic Manipulation. Proceedings of the 2021 26th IEEE International Conference on Emerging Technologies and Factory Automation (ETFA).

[22] Song Z., Yin J., Wang Z., Lu C., Yang Z., Zhao Z., Lin Z., Wang J., Wu C., Cheng J. (2022). A flexible triboelectric tactile sensor for simultaneous material and texture recognition. Nano Energy.

[23] Von Drigalski F., Gall M., Cho S.-G., Ding M., Takamatsu J., Ogasawara T., Asfour T. Textile identification using fingertip motion and 3D force sensors in an open-source gripper. Proceedings of the 2017 IEEE International Conference on Robotics and Biomimetics (ROBIO).

[24] Huang S., Wu H. (2021). Texture recognition based on perception data from a bionic tactile sensor. Sensors.

[25] Lima B.M.R., de Oliveira T.E.A., da Fonseca V.P. Classification of Textures using a Tactile-Enabled Finger in Dynamic Exploration Tasks. Proceedings of the 2021 IEEE Sensors.

[26] Weber A.I., Saal H.P., Lieber J.D., Cheng J.-W., Manfredi L.R., Dammann J.F., Bensmaia S.J. (2013). Spatial and temporal codes mediate the tactile perception of natural textures. Proc. Natl. Acad. Sci. USA.

[27] Jenmalm P., Birznieks I., Goodwin A.W., Johansson R.S. (2003). Influence of object shape on responses of human tactile afferents under conditions characteristic of manipulation. Eur. J. Neurosci..

[28] Johansson R.S., Birznieks I. (2004). First spikes in ensembles of human tactile afferents code complex spatial fingertip events. Nat. Neurosci..

[29] Johansson R.S., Flanagan J.R. (2009). Coding and use of tactile signals from the fingertips in object manipulation tasks. Nat. Rev. Neurosci..

[30] Zhu P., Wang Y., Wang Y., Mao H., Zhang Q., Deng Y. (2020). Flexible 3D architectured piezo/thermoelectric bimodal tactile sensor array for E-skin application. Adv. Energy Mater..

[31] Luo Y., Xiao Q., Li B. (2017). A stretchable pressure-sensitive array based on polymer matrix. Sensors.

[32] Lin W., Wang B., Peng G., Shan Y., Hu H., Yang Z. (2021). Skin-inspired piezoelectric tactile sensor array with crosstalk-free row+ column electrodes for spatiotemporally distinguishing diverse stimuli. Adv. Sci..

[33] Slepyan A., Sankar S., Thakor N. Texture discrimination using a neuromimetic asynchronous flexible tactile sensor array with spatial frequency encoding. Proceedings of the 2021 10th International IEEE/EMBS Conference on Neural Engineering (NER).

[34] Gupta A.K., Nakagawa-Silva A., Lepora N.F., Thakor N.V. (2021). Spatio-temporal encoding improves neuromorphic tactile texture classification. IEEE Sens. J..

[35] Dargahi J., Najarian S. (2004). Human tactile perception as a standard for artificial tactile sensing—A review. Int. J. Med. Robot. Comput. Assist. Surg..

[36] Gong Y., Cheng X., Wu Z., Liu Y., Yu P., Hu X. (2021). A flexible tactile sensor array for dynamic triaxial force measurement based on aligned piezoresistive nanofibers. IEEE Sens. J..

[37] Yu P., Liu W., Gu C., Cheng X., Fu X. (2016). Flexible piezoelectric tactile sensor array for dynamic three-axis force measurement. Sensors.

[38] Wang Y., Ding W., Mei D. (2021). Development of flexible tactile sensor for the envelop of curved robotic hand finger in grasping force sensing. Measurement.

[39] Lee K.-W., Kim S.-C., Lim S.-C. (2022). DeepTouch: Enabling Touch Interaction in Underwater Environments by Learning Touch-Induced Inertial Motions. IEEE Sens. J..

[40] Lee D.-H., Hwang W., Lim S.-C. (2018). Interaction force estimation using camera and electrical current without force/torque sensor. IEEE Sens. J..

[41] Ko D.-K., Lee K.-W., Lee D.H., Lim S.-C. (2023). Vision-based interaction force estimation for robot grip motion without tactile/force sensor. Expert Syst. Appl..

[42] De Vignemont F., Ehrsson H.H., Haggard P. (2005). Bodily illusions modulate tactile perception. Curr. Biol..

[43] Yoshioka T., Craig J.C., Beck G.C., Hsiao S.S. (2011). Perceptual constancy of texture roughness in the tactile system. J. Neurosci..

[44] Zhao X., Zhang Z., Xu L., Gao F., Zhao B., Ouyang T., Kang Z., Liao Q., Zhang Y. (2021). Fingerprint-inspired electronic skin based on triboelectric nanogenerator for fine texture recognition. Nano Energy.

[45] Sinapov J., Sukhoy V., Sahai R., Stoytchev A. (2011). Vibrotactile recognition and categorization of surfaces by a humanoid robot. IEEE Trans. Robot..

[46] Zhengkun Y., Yilei Z. (2017). Recognizing tactile surface roughness with a biomimetic fingertip: A soft neuromorphic approach. Neurocomputing.

[47] Baishya S.S., Bäuml B. Robust material classification with a tactile skin using deep learning. Proceedings of the 2016 IEEE/RSJ International Conference on Intelligent Robots and Systems (IROS).

[48] Chun S., Choi Y., Suh D.I., Bae G.Y., Hyun S., Park W. (2017). A tactile sensor using single layer graphene for surface texture recognition. Nanoscale.

[49] Friedl K.E., Voelker A.R., Peer A., Eliasmith C. (2016). Human-inspired neurorobotic system for classifying surface textures by touch. IEEE Robot. Autom. Lett..

[50] Hochreiter S., Schmidhuber J. (1997). Long short-term memory. Neural Comput..

[51] Yu Y., Si X., Hu C., Zhang J. (2019). A review of recurrent neural networks: LSTM cells and network architectures. Neural Comput..

[52] Altché F., de La Fortelle A. An LSTM network for highway trajectory prediction. Proceedings of the 2017 IEEE 20th International Conference on Intelligent Transportation Systems (ITSC).

[53] Qu Z., Haghani P., Weinstein E., Moreno P. Syllable-based acoustic modeling with CTC-SMBR-LSTM. Proceedings of the 2017 IEEE Automatic Speech Recognition and Understanding Workshop (ASRU).

[54] Hsu W.-N., Zhang Y., Lee A., Glass J. Exploiting depth and highway connections in convolutional recurrent deep neural networks for speech recognition. Proceedings of the INTERSPEECH 2016.

[55] Ramachandran P., Zoph B., Le Q.V. (2017). Searching for activation functions. arXiv.

[56] Kingma D.P., Ba J. (2014). Adam: A method for stochastic optimization. arXiv.

[57] Overvliet K., Smeets J.B., Brenner E. (2008). The use of proprioception and tactile information in haptic search. Acta Psychol..

[58] Klatzky R.L., Lederman S.J., Reed C. (1987). There’s more to touch than meets the eye: The salience of object attributes for haptics with and without vision. J. Exp. Psychol. Gen..

[59] Blake D.T., Hsiao S.S., Johnson K.O. (1997). Neural coding mechanisms in tactile pattern recognition: The relative contributions of slowly and rapidly adapting mechanoreceptors to perceived roughness. J. Neurosci..

[60] Smith A.M., Chapman C.E., Deslandes M., Langlais J.-S., Thibodeau M.-P. (2002). Role of friction and tangential force variation in the subjective scaling of tactile roughness. Exp. Brain Res..

